# Nano‐Enabled Reposition of Proton Pump Inhibitors for TLR Inhibition: Toward A New Targeted Nanotherapy for Acute Lung Injury

**DOI:** 10.1002/advs.202104051

**Published:** 2021-11-23

**Authors:** Liya Sun, Yuan Liu, Xiali Liu, Rui Wang, Jiameng Gong, Aabida Saferali, Wei Gao, Aying Ma, Huiqiang Ma, Stuart E. Turvey, Shan‐Yu Fung, Hong Yang

**Affiliations:** ^1^ School of Biomedical Engineering The Province and Ministry Co‐Sponsored Collaborative Innovation Center for Medical Epigenetics Intensive Care Unit of the Second Hospital Tianjin Medical University No. 22 Qixiangtai Road, Heping District Tianjin 300070 China; ^2^ Department of Pulmonary and Critical Care Medicine Shanghai General Hospital Shanghai Jiao Tong University School of Medicine No. 650 Xinsongjiang Road Shanghai 201620 China; ^3^ Channing Division of Network Medicine Brigham and Women's Hospital Harvard Medical School 181 Longwood Avenue Boston MA 02115 USA; ^4^ BC Children's Research Institute University of British Columbia 950 West 28th Avenue Vancouver BC V5Z 4H4 Canada; ^5^ Department of Immunology Key Laboratory of Immune Microenvironment and Disease (Ministry of Education) The Province and Ministry Co‐Sponsored Collaborative Innovation Center for Medical Epigenetics School of Basic Medical Science Tianjin Medical University No. 22 Qixiangtai Road, Heping District Tianjin 300070 China

**Keywords:** acute lung injury, bioactive nanoparticles, drug repurposing, inflammation, macrophages, proton pump inhibitors, Toll‐like receptors

## Abstract

Toll‐like receptor (TLR) activation in macrophages plays a critical role in the pathogenesis of acute lung injury (ALI). While TLR inhibition is a promising strategy to control the overwhelming inflammation in ALI, there still lacks effective TLR inhibitors for clinical uses to date. A unique class of peptide‐coated gold nanoparticles (GNPs) is previously discovered, which effectively inhibited TLR signaling and protected mice from lipopolysaccharide (LPS)‐induced ALI. To fast translate such a discovery into potential clinical applicable nanotherapeutics, herein an elegant strategy of “nano‐enabled drug repurposing” with “nano‐targeting” is introduced to empower the existing drugs for new uses. Combining transcriptome sequencing with Connectivity Map analysis, it is identified that the proton pump inhibitors (PPIs) share similar mechanisms of action to the discovered GNP‐based TLR inhibitor. It is confirmed that PPIs (including omeprazole) do inhibit endosomal TLR signaling and inflammatory responses in macrophages and human peripheral blood mononuclear cells, and exhibits anti‐inflammatory activity in an LPS‐induced ALI mouse model. The omeprazole is then formulated into a nanoform with liposomes to enhance its macrophage targeting ability and the therapeutic efficacy in vivo. This research provides a new translational strategy of nano‐enabled drug repurposing to translate bioactive nanoparticles into clinically used drugs and targeted nano‐therapeutics for ALI.

## Graphical abstract

A new concept of “nano‐enabled drug repurposing” is proposed based on understanding the nanoparticle‐immune system interactions to empower the existing drugs for new uses. Herein, the proton pump inhibitors are identified to share similar mechanisms of action to bioactive gold nanoparticles with undiscovered Toll‐like receptor inhibitory and anti‐inflammatory activities, facilitating the translation of bioactive nanoparticles into clinically applicable nanotherapeutics.

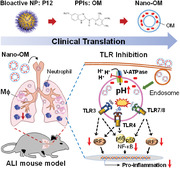

## 1 Introduction

Acute respiratory distress syndrome (ARDS) or its less severe form of acute lung injury (ALI) are serious and devastating conditions of critically ill patients with clinical features including acute hypoxemic respiratory failure and bilateral pulmonary infiltrates.^[^ ^1^ ^]^ Although significant progress has been made in understanding the pathogenesis of ARDS since its first report in 1967,^[^ ^2^ ^]^ there still lacks an effective pharmacotherapy due to the complexity of the syndrome. The only effective therapy to reduce its mortality is the small tidal volume ventilation.^[^ ^3^ ^]^ Currently, the mortality rate of ARDS is still high, ranging from 35% to 46%,^[^ ^4^ ^]^ so it is urgently needed to find new clinically available agents to treat ALI/ARDS.

The overwhelming inflammatory reactions in the lungs are the hallmark of ALI/ARDS pathogenesis. The outbreaks of inflammation in the early stage of ALI/ARDS have largely been attributed to the macrophages in the lung through the activation of their pattern recognition receptors, especially Toll‐like receptors (TLRs).^[^ ^5^ ^]^ Therefore, targeting lung macrophages to precisely regulate the activation of TLR signaling may serve as an effective strategy to control the early inflammatory responses in ALI/ARDS.

There are many compounds and devices developed to intervene TLR signaling pathways, including small molecule inhibitors, antibodies, oligonucleotides, lipid A analogs, microRNAs, and nanodevices.^[^ ^6^ ^]^ Despite extensive efforts have been made in developing these effective TLR inhibitors, none are successful for clinical uses. Therefore, it is important to search for new, potent TLR inhibitors that can fulfill clinical needs. Accordingly, the nanoform of TLR inhibitors is emerging as a novel class of nano‐therapies owing to their desired pharmacological properties in cell targeting and tissue‐distribution.^[^ ^7^ ^]^

We previously discovered a new class of peptide‐gold nanoparticle (GNP) hybrids‐P12 that could effectively inhibit multiple TLR signaling pathways and reduce inflammation through regulating the endosomal acidification process in macrophages.^[^ ^8^ ^]^ However, the core of this nanodrug is made of non‐biodegradable GNPs, which limits the clinical translation of P12. To tackle this problem, one idea was to seek clinically used drugs that have similar working mechanisms as P12, and repurpose their use for ALI/ARDS. This “nano‐enabled drug repurposing” can be done by taking advantage of the rapid advancement of 2nd generation gene sequencing with bioinformatics analysis to obtain the genomic signature (altered gene expression pattern) of P12 in comparison with those of over 1300 bioactive small molecules treated human cells by the Connectivity Map (also known as “CMAP”).^[^ ^9^ ^]^ This approach allows the discovery of the relationships between the therapeutic agents, gene expression changes, and biological pathways to repurpose the “old drugs” for new uses.

In this study, we first applied CMAP analysis based on the P12 altered gene expression profiles on human peripheral blood mononuclear cells (PBMC), and identified the “old drugs” proton pump inhibitors (PPIs) that have similar genomic signatures as P12. We then evaluated several clinically used PPIs for their inhibitory capability on TLR4 signaling in vitro. Using omeprazole (OM) as a representative PPI, we found that OM could potently inhibit multiple endosomal TLRs, including TLR3, TLR4, and TLR7/8, and reduce the downstream pro‐inflammatory cytokine production. The novel activities of OM in TLR inhibition and anti‐inflammation were also confirmed by RNA‐seq analysis. In order to enable the targeting ability of OM to lung macrophages, OM was formulated into liposomes (namely Nano‐OM); its effectiveness in TLR inhibition and anti‐inflammatory activity was then verified in vitro. Next, a lipopolysaccharide (LPS)‐induced ALI mouse model was employed to evaluate the therapeutic effect of Nano‐OM on reducing lung inflammation and injury upon intratracheal instillation. This study transformed non‐biodegradable nanoparticles with therapeutic potential into new targeted clinically applicable nanomedicine, providing a new strategy for rapid development of targeted nano‐therapies for ALI/ARDS.

## 2 Results

### 2.1 Identification of PPIs as a New Class of Anti‐Inflammatory Agents Resembling P12

**Figure 1:**
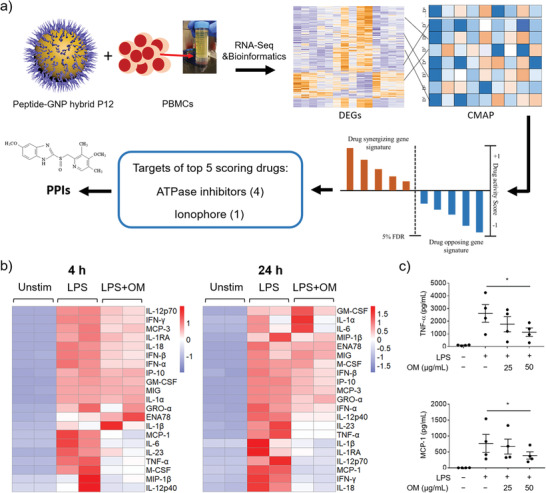
Identification of PPIs as drug candidates that shared a similar mechanism of anti‐inflammation to P12 by CMAP analysis. a) Top 5 compounds are identified to have a similar mechanism to P12 from RNA‐seq and CMAP analysis; DEGs: differentially expressed genes. b) The heatmap of cytokine profiles of PBMC stimulated with LPS (10 ng mL^−1^) for 4 and 24 h with/without OM (50 µg mL^−1^) treatment from a multiplexed Luminex assay. c) Selected cytokines (TNF‐*α* and MCP‐1) expression in PBMC with LPS stimulation for 4 h in the presence of OM (25, 50 µg mL^−1^) confirmed by ELISA; *N* = 4, *p < 0.05.

We previously developed a novel class of anti‐inflammatory nanoparticles (namely P12) made of a hexapeptide coating on a 13‐nm GNP core.^[^ ^8b^ ^]^ It was found that P12 exhibited potent inhibitory activity on TLR signaling via modulating the endosomal pH in macrophages.^[^ ^8a^ ^]^ Using the RNA sequencing technique, we collected the global gene expression profiles of P12 treatment in comparison with LPS stimulation in human PBMC.^[^ ^8a^ ^]^ With this set of differentially expressed genes, we applied CMAP analysis to search for the best match between the differentially expressed gene set of P12 and the genomic profiles of over 1300 drugs for synergizing gene signatures, and ranked these drugs by score (**Figure** **1**a). The top 5 candidate drugs with the highest absolute scores were ATPase inhibitors and ionophore (Figure 1a). The identified ionophore was monensin, a well‐known drug to inhibit the acidification of endosome‐lysosome system,^[^ ^10^ ^]^ which shares a similar mechanism of action of P12. This reminded us of the clinically commonly used PPIs, which can irreversibly block the H^+^/K^+^ ATPase in gastric parietal cells to control the acid production in the stomach, and are commonly prescribed to treat gastroesophageal reflux and peptic ulcer diseases.^[^ ^11^ ^]^ In addition, PPIs are able to inhibit vacuolar H^+^‐ATPase (V‐ATPase),^[^ ^12^ ^]^ which is responsible for the acidification of the intracellular compartments, including the endosomes and lysosomes, to increase the endosomal/lysosomal pH.^[^ ^13^ ^]^ Because PPIs and P12 share a similar working mechanism on regulating vesicular pH, we hypothesized that PPIs may also be able to inhibit TLR signaling through blocking endosomal/lysosomal acidification and exhibit anti‐inflammatory activity.

To test this hypothesis, we selected one of the commonly used PPIs in clinics, OM, to validate its ability of TLR signaling inhibition and anti‐inflammatory activity. Using a 21‐plexed cytokine assay, we found that OM could inhibit the production of multiple chemokines and cytokines, including TNF‐*α*, MCP‐1, IL‐23, and IL‐12 (Figure 1b), upon LPS stimulation for 4 and 24 h in human PBMC from two healthy donors. Selected cytokines (TNF‐*α* and MCP‐1) were verified by ELISA with four different donors (Figure 1c). Note that the viability of PBMC remained unchanged by OM treatment (Figure S1, Supporting Information). These results clearly showed that OM could inhibit a variety of LPS‐induced pro‐inflammatory cytokines production in PBMC, suggesting that OM indeed has anti‐inflammatory activity.

**Figure 2:**
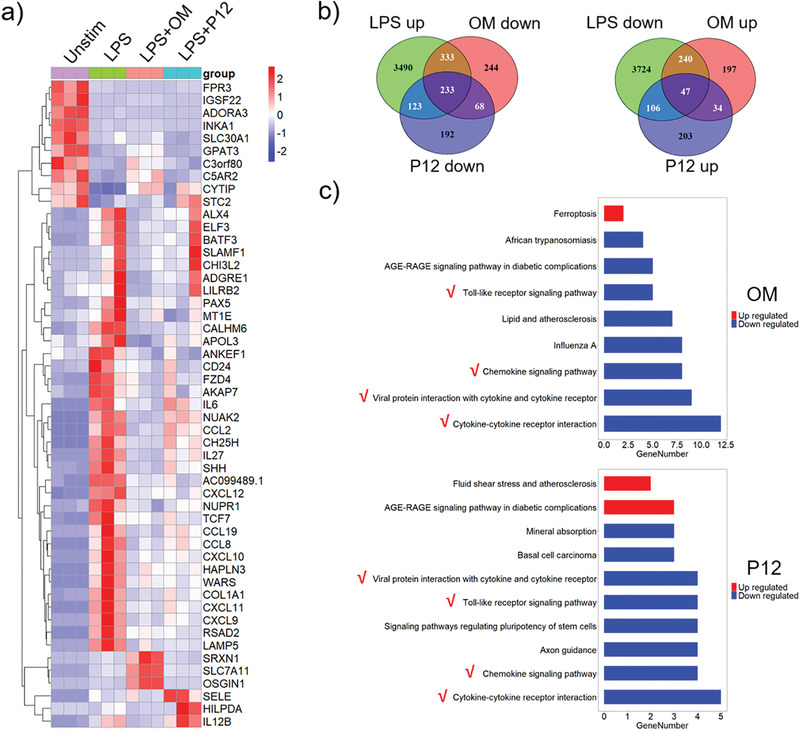
The gene expression and signaling pathways regulated by OM and P12 in THP‐1 cell‐derived macrophages under LPS stimulation by RNA‐seq analysis. a) The heatmap of top 51 differentially expressed genes of 4 study groups: Unstim, LPS, LPS+OM, and LPS+P12; *p* < 0.05 and log2 (fc) > 1 are set as the threshold. b) The Venn diagram of LPS responsive genes that are up‐regulated or down‐regulated by OM and P12 treatments in THP‐1 cell‐derived macrophages; *p* < 0.05 and |log2 (fc)| > 0 are used as the cut‐off criteria. c) Statistical enrichment of differential expression genes in KEGG pathways for OM and P12 treatment. The red check marks indicate the pathways that are affected by both OM and P12. All pathways shown are significantly enriched with *p* < 0.05, *N* = 3, LPS = 10 ng mL^−1^, OM = 50 µg mL^−1^, P12 = 100 nM.

Next, we analyzed the altered gene clusters and signal pathways shared by OM and P12 in THP‐1 cell‐derived macrophages. Cells were stimulated by LPS and treated with OM and P12 for 4 h, followed by RNA‐seq transcriptome analysis. The differentially expressed genes (top 51) in Unstim, LPS, LPS+OM, and LPS+P12 groups were shown in the heatmap (**Figure** **2**a). As shown in the Venn diagram (Figure 2b), a total of 8296 genes expression was altered upon LPS stimulation, of which 4179 genes were up‐regulated and 4117 genes were down‐regulated. Among the up‐regulated genes by LPS, OM and P12 treatment inhibited the expression of 566 and 356 genes, respectively (Figure 2b left), where 233 genes were down‐regulated by both OM and P12. For the LPS down‐regulated genes, OM and P12 treatment increased the expression of 287 and 153 genes, respectively (Figure 2b right), where only 47 genes altered by both. The Kyoto Encyclopedia of Genes and Genomes (KEGG) pathway database analysis revealed that OM and P12 affected same key signal pathways in relation to inflammation, such as TLR signaling pathway, chemokine signaling pathway, viral protein interaction with cytokine and cytokine receptor, and the cytokine‐cytokine receptor interaction (Figure 2c). Thus, at the transcriptomic level, OM was able to regulate inflammatory responses in a way similar to P12.

### 2.2 The Inhibition of TLR4 Signaling by PPIs

**Figure 3:**
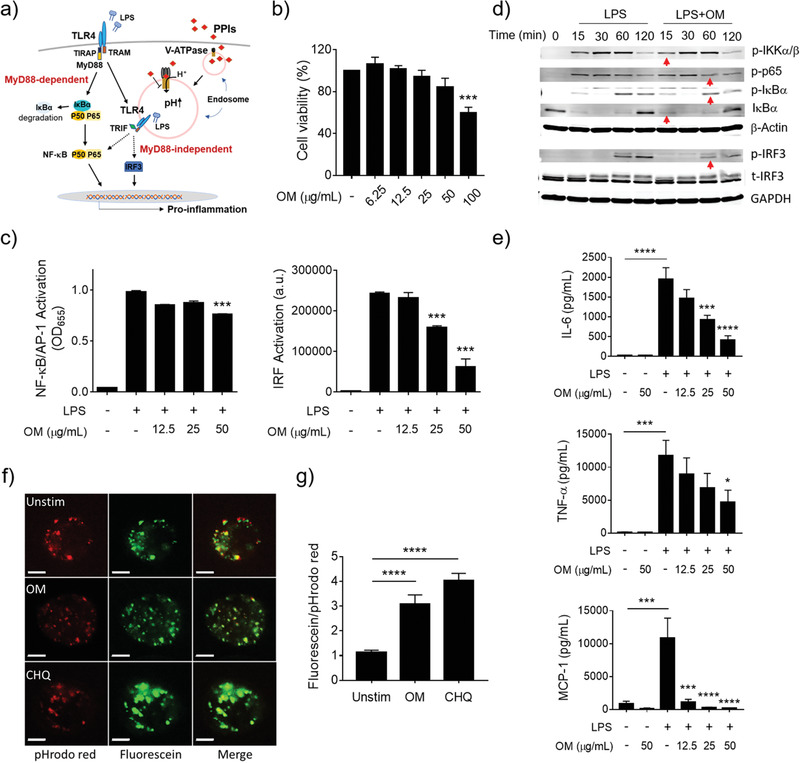
OM inhibited TLR4 signaling and the endosomal acidification in the THP‐1 cell‐derived macrophages. a) A scheme showing the inhibitory effect of PPIs (OM) on the TLR4 signaling pathway. b) Viability of THP‐1 cell‐derived macrophages treated with various concentrations of OM (*N* = 5). c) The concentration‐dependent inhibitory effects of OM on the activation of NF‐*κ*B/AP‐1 (left) and IRF (right) triggered by LPS (10 ng mL^−1^). d) Immunoblotting validating the inhibition of OM on the phosphorylation of IKK*α*/*β* (p‐IKK*α*/*β*), p65 (p‐p65), I*κ*B*α* (p‐I*κ*Β*α*), and degradation of I*κ*Β*α* for NF‐*κ*B activation, and the phosphorylation of IRF3 (p‐IRF3) for IRF activation. e) Inhibition of cytokine production (IL‐6, TNF‐*α*, and MCP‐1) by THP‐1 cell‐derived macrophages stimulated by LPS with the presence or absence of various concentrations of OM, *N* = 5 for TNF‐*α*. f) Confocal microscopic images of THP‐1 cell‐derived macrophages treated with OM (500 µg mL^−1^) and chloroquine (CHQ, 30 µM); the scale bar represents 5 µm; the endosomal pH is probed by pHrodo red (red) and fluorescein (green) labeled dextran. g) The quantification of the green‐to‐red ratio of the fluorescence signals from 32 to 36 cells. *N* = 3 unless specified, *p < 0.05, **p < 0.01, ***p < 0.001, ****p < 0.0001.

To further verify whether OM could inhibit TLR signaling, we employed the nuclear factor‐*κ*B/activator protein 1 (NF‐*κ*B/AP‐1) and interferon regulatory factor (IRF) reporter cell systems to evaluate the effects of OM on the two arms of TLR4 signaling, the myeloid differentiation factor 88 (MyD88)‐dependent and MyD88‐independent signaling pathways, respectively (**Figure** **3**a). First, we confirmed that OM did not have toxicity to the THP‐1 cell‐derived macrophages up to 50 µg mL^−1^ for 24 h (Figure 3b); however, at a higher concentration of 100 µg mL^−1^, OM exhibited some toxicity to macrophages as the cell viability significantly decreased to 57%. Accordingly, the OM concentration of 50 µg mL^−1^ was used for the rest of the study. Using the reporter cells, we found that OM significantly inhibited both NF‐*κ*B/AP‐1 (left) and IRF (right) activation triggered by LPS (10 ng mL^−1^), and the inhibitory effect was stronger on the IRF activation than on the NF‐*κ*B activation (Figure 3c). This inhibitory effect was also confirmed by directly looking at phosphorylation of the inhibitor of NF‐*κ*B (I*κ*B) kinase *α*/*β* (IKK*α*/*β*) (p‐IKK*α*/*β*), p65 (p‐p65), and I*κ*B*α* (p‐I*κ*B*α*) as well as the degradation of I*κ*B*α* for NF‐*κ*B activation, and the phosphorylation of IRF3 (p‐IRF3) for IRF3 activation (Figure 3d). In addition, OM treatment also inhibited the pro‐inflammatory cytokine production (interluekin‐6 (IL‐6), tumor necrosis factor *α* (TNF‐*α*), and monocyte chemoattractant protein‐1 (MCP‐1)) induced by LPS in a dose‐dependent manner (Figure 3e), confirming the anti‐inflammatory activity of OM in macrophages.

As OM was expected to have a similar mechanism as P12 on the modulation of endosomal pH, we applied two pH‐sensitive fluorescence probes, pHrodo red and fluorescein‐labeled dextrans, to observe the changes in endosomal pH upon OM treatment. The increase in the ratio of the fluorescence intensity of fluorescein to pHrodo red indicates the elevation of pH in the endosomal compartment.^[^ ^8^, ^14^ ^]^ The classic endosomal pH modulator chloroquine, capable of preventing endosomal acidification, served as the positive control. As shown in Figure 3f, the red color (pHrodo red) became dimmer in the chloroquine and OM treated groups compared with the untreated cells; the ratio of green to red fluorescence significantly increased in both chloroquine and OM treated cells compared with untreated group (Figure 3g), indicating an increase in endosomal pH. These results suggested that OM treatment could prevent the endosomal acidification.

It should be noted that the observed TLR4 inhibition was universal for the same class of PPIs including OM and other three clinically used pantoprazole, lansoprazole, and rabeprazole. Similar to OM, the other three PPIs were capable of inhibiting the activation of NF‐*κ*B and IRF in TLR4 signaling pathways as well (Figure S2, Supporting Information).

### 2.3 Inhibition of OM on the Endosomal TLR Pathways

**Figure 4:**
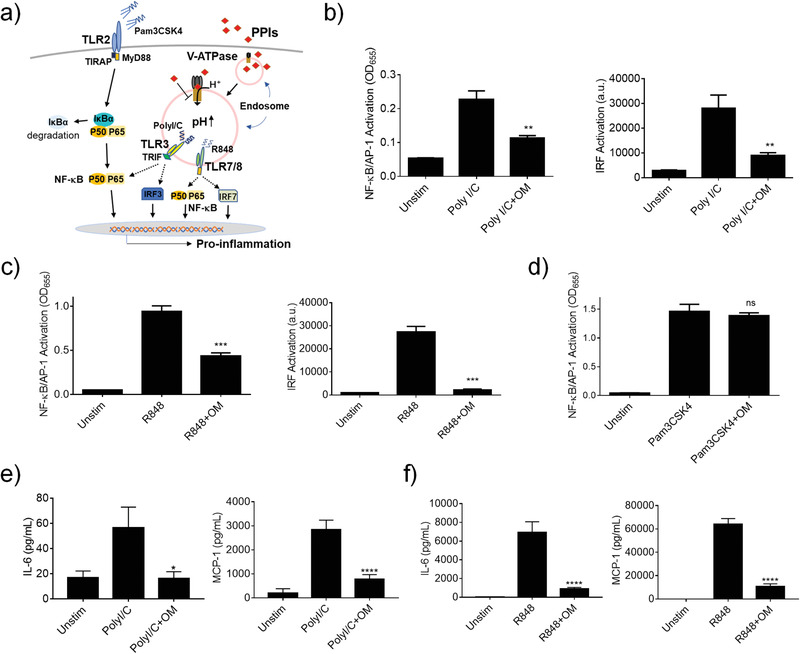
Inhibitory activity of OM on TLR3 and TLR7/8 signaling pathways in THP‐1 cell‐derived macrophages. a) A scheme demonstrating the inhibitory effect of the PPIs on endosomal TLR activation. OM significantly inhibited NF‐*κ*B/AP‐1 (left) and IRF (right) activation stimulated by b) PolyI/C (50 µg mL^−1^) on TLR3 and c) R848 (10 µg mL^−1^) on TLR7/8 signaling. d) OM could not inhibit NF‐*κ*B/AP‐1 activation induced by Pam3CSK4 (10 ng mL^−1^, TLR2 signaling). Inhibition of IL‐6 and MCP‐1 production by OM upon e) PolyI/C and f) R848 stimulation. OM = 50 µg mL^−1^, N = 3, ns: not significant, *p < 0.05, **p < 0.01, ***p < 0.001, ****p < 0.0001.

Since OM was found to mainly modulate the acidification process in endosomes, we anticipated that the inhibitory activity of OM would likely be more specific to the endosomal TLR signaling (**Figure** **4**a). Among 9 human TLRs with well‐known function, TLRs 1, 2, 5, and 6 are mainly found on the cell surface, while TLRs 3, 7, 8, and 9 are primarily expressed in the endosomal compartments;^[^ ^15^ ^]^ TLR4 can localize both on the cell surface and in the endosomes. Using different TLR agonists, we found that OM was able to inhibit the activation of both NF‐*κ*B/AP‐1 and IRF on TLR3 (activated by PolyI/C) and TLR7/8 (activated by R848) signaling (Figure 4b,c) but had no effect on Pam3CSK4 (TLR1/2 ligand) triggered activation of NF‐*κ*B/AP‐1 (Figure 4d). The inhibition of OM on endosomal TLRs was validated by measuring the downstream cytokine production, where the levels of IL‐6 and MCP‐1 secreted by THP‐1 cell‐derived macrophages were significantly reduced by OM under PolyI/C (Figure 4e) or R848 (Figure 4f) stimulation. Taken together, these results clearly showed that OM indeed could specifically attenuate endosomal TLR signaling to exert the anti‐inflammatory activity.

In addition to endosomal TLR signaling, we were also wondering if OM could attenuate other well‐known inflammatory pathways signaling from the cell surface membrane and endosomes. Tumor necrosis factor receptor (TNFR) (for TNF‐*α*) and interleukin‐1 receptor (IL‐1R) (for IL‐1*α* and IL‐1*β*) respond to signals from the cell surface,^[^ ^16^ ^]^ while the type I IFN receptor (IFNAR) can signal from endosomes.^[^ ^17^ ^]^ Interestingly, we found that OM did not affect the TNFR and IL‐1R signaling, but significantly inhibited IFN‐*β* induced activation of both NF‐*κ*B and IRF through IFNAR (Figure S3, Supporting Information). This suggested that OM could broadly affect endosomal signaling pathways including endosomal TLRs.

It is worth mentioning that the inhibitory activity of OM was not cell type specific. OM treatment could also significantly reduce IL‐6, MCP‐1, and IL‐8 production upon LPS stimulation in BEAS‐2B cells (airway epithelial cells) (Figure S4, Supporting Information) and inhibit PolyI/C‐induced activation of NF‐*κ*B and IRF in A549 reporter cells (lung epithelial cells) (Figure S5, Supporting Information).

### 2.4 OM Exhibited Anti‐Inflammatory Activity in LPS‐Induced ALI Mice

In the above studies, we have confirmed that OM was able to inhibit endosomal TLR signaling and regulate inflammatory responses in vitro. In order to repurpose its use in controlling acute lung inflammation, we investigated the effect of OM on an LPS‐induced ALI mouse model. As shown in Figure S6, Supporting Information, OM (at the dose of 10 mg kg^−1^ via intratracheal instillation) could effectively reduce lung inflammation by inhibiting inflammatory cell infiltration, especially neutrophiles, and decreasing the pro‐inflammatory cytokine IL‐1*β* production in the bronchoalveolar lavage fluid (BALF). The lung histological analysis revealed that OM was able to decrease lung injury indicated by reduced alveolar neutrophiles and alveolar septal thickening scores. It should be noted that OM itself at such a dose also resulted in elevated alveolar septal thickening in healthy mice. Considering the potential side effects of OM acting on modulating endosomal acidification in various cell types, a targeted OM treatment may be more suitable and safer for ALI/ARDS.

### 2.5 Nano‐OM Fabrication and Inhibition on TLR4 Signaling Pathway

**Figure 5:**
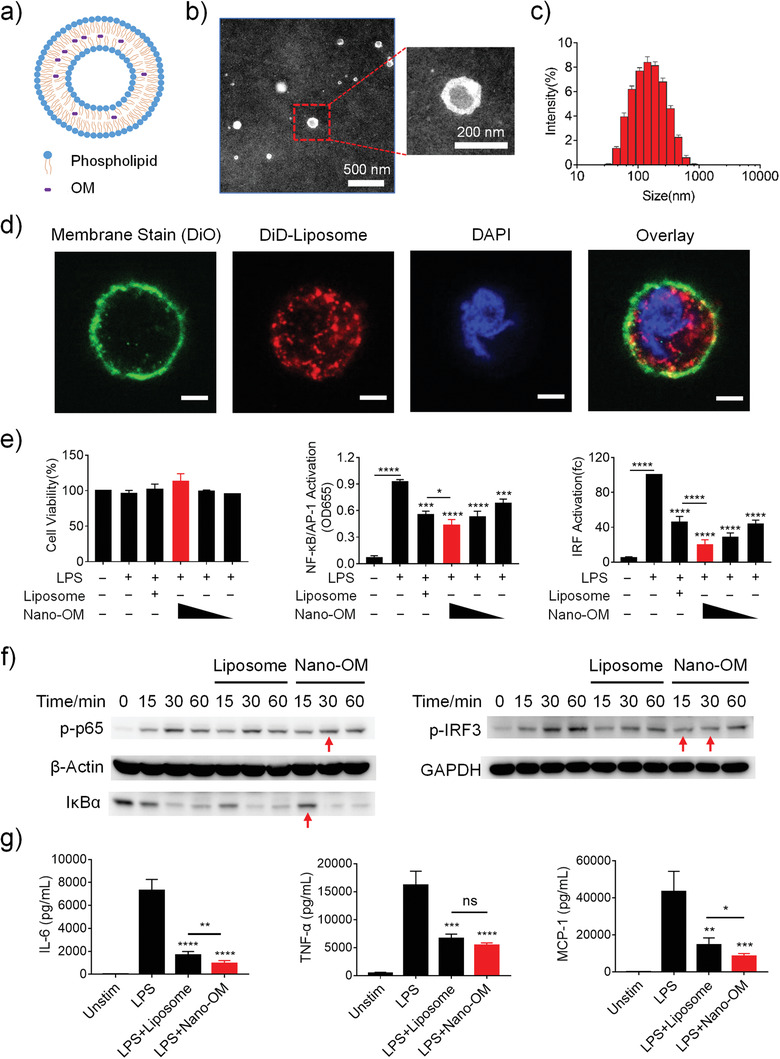
Fabrication of Nano‐OM and its anti‐inflammatory effect in THP‐1 cell‐derived macrophages. a) A schematic diagram of Nano‐OM. b) Transmission electron microscopy images of Nano‐OM. c) Size distribution of Nano‐OM by dynamic light scattering measurement. d) Confocal images of THP‐1 cell‐derived macrophages treated with DiD‐labeled liposomes (DiD‐Liposome); the cell membrane and nucleus are labeled with DiO and DAPI, respectively; the scale bar represents 5 µm. e) The effects of Nano‐OM on the viability (left) and the activation of NF‐*κ*B/AP‐1 (middle) and IRF (right) of the THP‐1 reporter cell‐derived macrophages upon LPS stimulation, *N* = 3; Nano‐OM (OM: 30, 15, 7.5 µg mL^−1^), Liposome (phospholipids: 5, 2.5, 1.25 mg mL^−1^). f) The inhibitory effect of Nano‐OM on the phosphorylation of p65 (p‐p65) and degradation of I*κ*B*α* (left) as well as the phosphorylation of IRF3 (p‐IRF3) of THP‐1 cell‐derived macrophages stimulated by LPS; the red arrows indicate the reduced expression compared with the LPS group, *N* = 3. g) Inhibition of IL‐6, TNF‐*α* and MCP‐1 production by Nano‐OM in THP‐1 cell‐derived macrophages upon LPS stimulation, *N* = 8 for IL‐6, *N* = 7 for TNF‐*α*, *N* = 5 for MCP‐1. LPS = 10 ng mL^−1^, Liposome (phospholipids: 5 mg mL^−1^), Nano‐OM (OM: 30 µg mL^−1^), *p < 0.05, ** *p* < 0.01, ***p < 0.001, ****p < 0.0001.

In order to increase the targeting capability of OM and reduce its potential side effects, the clinically approved nanocarrier liposomes^[^ ^18^ ^]^ were employed to formulate OM into a nano‐form, namely Nano‐OM. Based on the hydrophobic nature of OM molecule, OM was embedded into the phospholipid bilayer of liposomes as shown in **Figure** **5**a. Transmission electron microscopy images revealed that Nano‐OM had a spherical shape (Figure 5b), and its hydrodynamic size was found to be 144.9 ± 1.1 nm with a PDI value of 0.22 ± 0.01 by dynamic light scattering measurements (Figure 5c). The nano‐formulated OM was found to be effectively taken up by THP‐1 cell‐derived macrophages (Figure 5d). Using the same reporter cell systems, we found that Nano‐OM was able to inhibit LPS‐induced activation of NF‐*κ*B/AP‐1 and IRF in THP‐1 cell‐derived macrophages without affecting the cell viability (Figure 5e). Interestingly, liposomes showed some inhibitory effects; nevertheless, Nano‐OM exhibited stronger inhibitory activity at the concentration of 30 µg mL^−1^ than the liposome carrier control. Again, the inhibition was verified by direct analysis of NF‐*κ*B and IRF activation at the protein levels (Figure 5f), where the LPS‐induced phosphorylation of p65 and IRF3 as well as the degradation of I*κ*B*α* were decreased by Nano‐OM; the liposomes seemed to have some effects on p‐IRF3 as well. Furthermore, Nano‐OM was able to reduce the production of inflammatory cytokines IL‐6, TNF‐*α*, and MCP‐1 triggered by LPS stimulation (Figure 5g). As expected, Nano‐OM could also attenuate TLR3 and TLR7/8 signaling pathways (Figure S7, Supporting Information). Taken together, these results demonstrated that the nano‐formulated OM remained the effectiveness in regulating TLR signaling and associated inflammatory responses in macrophages.

### 2.6 The Therapeutic Activity of Nano‐OM in an LPS‐Induced ALI Mouse Model

**Figure 6:**
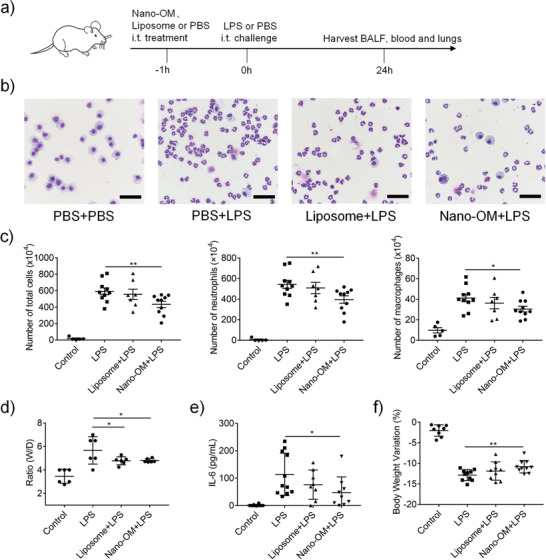
The anti‐inflammatory activity of Nano‐OM in the LPS‐induced ALI mouse model. a) The scheme of the LPS‐induced ALI model. b) Images of the BALF cells collected from four experimental groups. c) The total number of cells (left), neutrophil counts (middle), and macrophages counts (right) in the BALF. Nano‐OM reduced d) the lung W/D ratio and e) the IL‐6 level in the serum of ALI mice. f) Nano‐OM decreased the percentage loss of body weight variation in ALI mice. LPS = 10 mg kg^−1^, Nano‐OM: 75 µg kg^−1^ OM and 12.6 mg kg^−1^ phospholipids, liposome: 12.6 mg kg^−1^ phospholipids, *N* ≥ 5 per group, *p < 0.05, **p < 0.01, ***p < 0.001.

Since the TLR activation in alveolar macrophages plays an important role in the inflammatory responses and pathogenesis of ALI/ARDS, it is anticipated that Nano‐OM could be a novel targeted anti‐inflammatory nanodrug to control lung inflammation in ALI/ARDS. To verify this hypothesis, we first evaluated the therapeutic efficacy of Nano‐OM using a classical LPS induced ALI mouse model (**Figure** **6**a), where Nano‐OM (75 µg kg^−1^) was administered 1 h before LPS (10 mg kg^−1^) challenge through intratracheal injection, and the BALF, lungs and blood samples were collected 24 h later for various analyses. We found that mice with LPS challenge had significantly elevated total inflammatory cells in the BALF, whereas Nano‐OM treatment was able to reduce the total cell counts as well as the number of neutrophils and macrophages in the BALF (Figure 6b,c); note that liposomes alone had no effects on inhibiting the cell infiltration. Both Nano‐OM and liposomes were capable of reducing the ratio of wet to dry lung (W/D ratio) (Figure 6d) as the indication of the severity of pulmonary edema in ALI. Furthermore, the pro‐inflammatory cytokine IL‐6 level in the serum as well as the mouse body weight loss was reduced by Nano‐OM but not by liposomes (Figure 6e,f).

**Figure 7:**
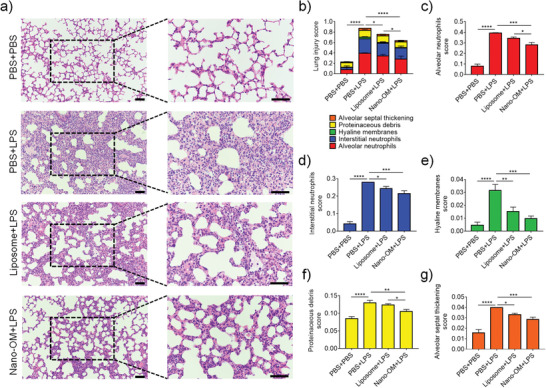
The protective effect of Nano‐OM on lung injury in LPS‐induced ALI mice. a) The histological images of H&E stained lung sections; the scale bar = 50 µm. b) Total lung injury score obtained from 5 pathophysiological characteristics based on the histological images. c) Alveolar neutrophils score. d) Interstitial neutrophils score. e) Hyaline membranes score. f) Proteinaceous debris score. g) Alveolar septal thickening score. LPS = 10 mg kg^−1^, Nano‐OM (OM: 75 µg kg^−1^; phospholipids: 12.6 mg kg^−1^), liposome (phospholipids: 12.6 mg kg^−1^), *N* = 6 per group, *p < 0.05, **p < 0.01, ***p < 0.001, ****p < 0.0001.

The histopathological analysis of ALI lungs also showed that Nano‐OM treatment was effective in decreasing lung inflammation and injury (**Figure** **7**). The severe inflammatory cell infiltration caused by LPS challenge could be inhibited by both Nano‐OM and liposome treatments (Figure 7a). The degree of LPS‐induced lung injury was further quantitatively assessed by scoring the five histological features of mouse lungs, including the alveolar neutrophils, interstitial neutrophils, hyaline membranes, proteinaceous debris, and alveolar septal thickening. As shown in Figure 7b, although Nano‐OM and liposome treatments were able to reduce the overall lung injury score, Nano‐OM appeared to be more effective than liposomes alone in ameliorating ALI, particularly on the alveolar and interstitial neutrophils score as well as the proteinaceous debris in the lungs (Figure 7c–g). All these results confirmed that Nano‐OM was effective to control lung inflammation and protect mice from ALI.

### 2.7 Targeting Capability of Nano‐OM to Pulmonary Macrophages

**Figure 8:**
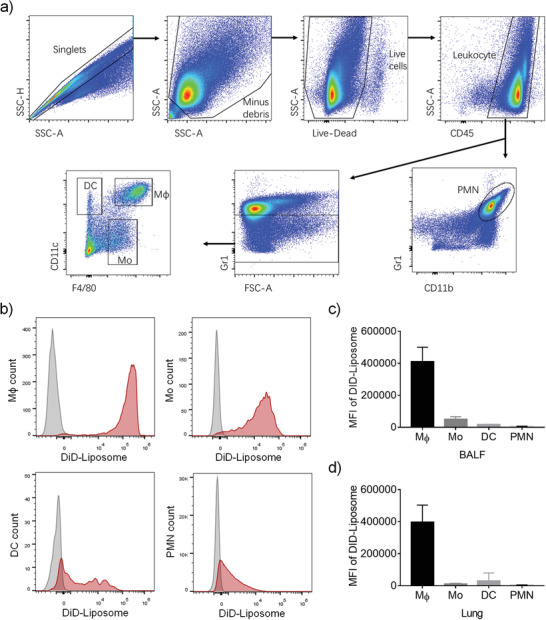
Nano‐OM targeted lung macrophages in ALI mice after intratracheal instillation. a) Gating strategy for the flow cytometry analysis to identify monocytes (Mo), dendritic cells (DCs), macrophages (M*ϕ*), and neutrophils (PMN) in the BALF. b) Distribution of DiD labeled liposomes (DiD‐Liposome) in various phagocytic immune cells; DiD‐Liposome treatment group is shown in red, and the untreated control group is shown in gray. Quantitative analysis of the amount of DiD‐Liposome internalized in various immune cells in c) the BALF and d) the lung; the uptake of DiD‐Liposome in each type of cells is quantified as the MFI of DiD. DiD‐Liposome: 12.6 mg kg^−1^ phospholipids, *N* = 4.

The purpose of formulating OM into Nano‐OM was to enhance its cellular targeting capability to macrophages. To verify this, we utilized a fluorescent dye, DiD, to label the liposomes and analyzed the preferential uptake of intratracheally administered DiD‐labeled liposomes in different cells in the BALF and the lung of ALI mice. The BALF cells were stained with fluorochrome‐conjugated antibodies to identify different immune cell types: macrophages (F4/80^+^CD11c^+^Gr1^−^), monocytes (F4/80^low^CD11c^−^Gr1^−^), dendritic cells (F4/80^−^CD11c^+^Gr1^−^), and neutrophils (Gr1^+^CD11b^+^) (**Figure** **8**a), followed by flow cytometry analysis. As expected, the DiD‐labeled liposomes could be taken up by these phagocytic immune cells (Figure 8b). By comparing the DiD mean fluorescence intensity (MFI) of these cell types, we found that DiD‐labeled liposomes were largely taken up by lung macrophages than by other three phagocytic immune cells in both the BALF and the lung single cell suspension (Figure 8c,d). These results suggested that the liposome formulated OM, Nano‐OM, mainly targeted lung macrophages upon intratracheal injection to attenuate lung inflammation of ALI mice.

## 3 Discussion

TLR signaling is essential in the host defense against infection. However, overactivation of TLRs can be harmful and has contributed to many inflammatory and autoimmune diseases. Therefore, TLR pathways have become attractive therapeutic targets for drug development. Despite great efforts have been put to develop specific inhibitors targeting different signaling steps in the TLR pathways, none have been approved for clinical use to date. Through repurposing the clinically used drugs, our study has identified a class of PPIs (e.g., OM) that have a similar mechanism of action (i.e., modulating endosomal acidification) to our previously developed purely materials‐based TLR nano‐inhibitors, the peptide‐coated GNPs (i.e., P12) without carrying any drug molecules. Like the peptide‐coated GNPs, PPIs can effectively inhibit endosomal TLR‐mediated inflammatory responses in vitro and in vivo. To further promote the targeting capability of PPIs to lung macrophages, we formulated OM with the liposomal nanocarrier into a nanoform, Nano‐OM. We confirmed that this new nanoform of OM exhibited effective anti‐inflammatory activity in vitro and in an ALI mouse model; more importantly, Nano‐OM revealed great targeting capability to lung macrophages.

### 3.1 Novel Anti‐Inflammatory Activity of PPIs by Inhibition of TLR Signaling Pathways

PPIs are a class of most commonly used drugs in clinics worldwide to control gastric acid secretion in the stomach for various gastrointestinal diseases.^[^ ^19^ ^]^ They can irreversibly bind and inhibit the H^+^/K^+^ ATPases that are expressed on parietal cells of the stomach.^[^ ^20^ ^]^ Additionally, PPIs can also inhibit the V‐ATPase,^[^ ^12^ ^]^ which pumps protons into intracellular vesicles such as endosomes and lysosomes through the plasma membrane in an ATP‐dependent manner in eukaryotic cells, subsequently leading to the acidification of these intracellular compartments.^[^ ^21^ ^]^ However, very few reports provide direct evidence referring PPIs act as TLR inhibitors.

Our results demonstrated that PPIs are able to effectively inhibit multiple TLR pathways including TLR3, TLR4, and TLR7/8 (Figures 1, 2, 3, 4). It has been found that the activation of endosomal TLRs (TLRs 3, 7/8, and 9) requires the acidification of endosomal/lysosomal compartments to cleave certain proteins and expose functional motifs for signaling transduction.^[^ ^22^ ^]^ This explains why PPIs are able to inhibit TLR3 and TLR7/8 but not the cell surface TLR2 (Figure 4). For TLR4, the activation of TRIF‐dependent signaling cascade is coupled with the TLR4 internalization and trafficking through endosomal/lysosomal compartments. This process involves the changes in the protein and lipid composition of the vesicle membrane components, which depends on the acidification of the lumen and the enrichment of hydrolytic enzymes for both the signal transduction and the degradation of TLR4.^[^ ^23^ ^]^ Indeed, studies have found that the internalization of TLR4 requires V‐ATPase‐mediated intra‐vesicular acidification.^[^ ^24^ ^]^ This also explains our observations that PPIs attenuated the two arms of TLR4 signaling cascades with a strong tendency toward IRF3 inhibition (Figure 3).

As the TLR activation can lead to the production of many pro‐inflammatory cytokines,^[^ ^25^ ^]^ TLR inhibition by PPIs will contribute to controlling the elevation of these inflammatory mediators, making PPIs novel anti‐inflammatory agents in addition to their conventional use. Like PPIs, drugs that are capable of preventing endosomal acidification, such as bafilomycin A1, ammonium chloride, and chloroquine, have been found to be able to abrogate the endosomal TLR signaling pathways as well.^[^ ^26^ ^]^ Thus, one could anticipate that the modulation of endosomal acidification process may represent a novel way to control TLR activation and regulate inflammation.

This novel anti‐inflammatory role of PPIs has been indirectly documented in a number of clinical observations. For example, patients with long‐term administration of PPIs after lung transplantation suffered less inflammatory complications in the grafts, independent of other clinical predictors, suggesting that PPIs may have some anti‐inflammatory effects.^[^ ^27^ ^]^ Experimentally, OM was found to alleviate hyperoxia‐induced lung injury in preterm rabbits;^[^ ^28^ ^]^ and lansoprazole could reduce the severity of rat enteritis induced by indomethacin.^[^ ^29^ ^]^ Although the specific mechanism behind the reported “anti‐inflammatory‐like” phenomena for PPIs is not clear, these observations strongly support our findings that PPIs can exert anti‐inflammatory activity by inhibiting TLR pathways, which retrospectively provides a possible explanation for the clinical observations.

### 3.2 The Advantages of Nano‐Enabled Drug Repurposing for New Clinical Applications

New drug development is a very long, high‐cost, and risky process before a drug can be put into the market. It has been estimated that the average cost for new drug development is about US$1000 million; ironically, the chance to fail in the development pipeline and clinical trials is extremely high.^[^ ^30^ ^]^ In view of this, seeking the new applications of listed old drugs has received much attention to quickly supply new therapies for clinical needs. Since the pharmacokinetics and safety profiles of existing drugs have been well established, the development process for their new applications can be accelerated into Phase II clinical evaluation.^[^ ^31^ ^]^ Accordingly, this can save about 40% of R&D costs and shorten the development cycle significantly to 3–12 years.^[^ ^32^ ^]^ Therefore, drug repurposing can certainly compensate for the shortcomings in current new drug development.

Nano‐biomaterials may serve as a novel strategy to spark the repurposing of old drugs for new medical applications. The rapid advances in nanotechnology have created diverse innovative nanomaterials that possess various surface chemistry and properties to tackle medical problems.^[^ ^33^ ^]^ Recent studies have discovered that nanoparticles by design with specific physicochemical properties can modulate immune responses (up‐ or down‐regulation) to exert therapeutic effects without carrying any drug payload.^[^ ^34^ ^]^ For example, the previously discovered peptide‐GNP hybrid P12, which is made of a 13‐nm GNP core coated with non‐bioactive hexapeptides, can effectively inhibit multiple TLR signaling pathways and reduce inflammation in macrophages.^[^ ^8a^ ^]^ Similarly, the “drug‐free” poly‐lactic‐co‐glycolic acid (PLGA) and poly‐lactic acid (PLA) nanoparticles when manufactured using the anionic surfactant poly‐ethylene‐alt‐maleic acid (PEMA) also exhibit significant inhibitory activities on TLR4 and TLR9 signaling in antigen‐presenting cells.^[^ ^35^ ^]^ Despite the exciting therapeutic activities of these novel nanodevices, their translation into clinical uses is often hindered as to the new drug development. Particularly, the biosafety issues of the nanomaterials‐based “drugs” need to be carefully addressed prior to the time‐consuming clinical trials. To get around this problem, we combined the RNA‐Seq technique with bioinformatic analysis of CMAP to identify existing drug compounds that share the similar mechanisms of action with the bioactive nanodevices. Such a “nano‐enabled drug repurposing” strategy is advantageous to new drug development. First, it allows the identification of unknown novel therapeutic activities for old drugs. Second, it expands the clinical applications of current drugs to treat different diseases, some of which have been suffering from the lack of effective drug therapies. Third, this approach dramatically saves the time and cost in the new drug development.

### 3.3 Potential Applications of Nano‐PPIs as a New Targeted Anti‐Inflammatory Therapy for the Treatment of ALI/ARDS

ALI/ARDS is still a severe disease with high mortality in the intensive care unit. Current treatments rely primarily on supportive interventions (i.e., mechanical ventilation), and there have been no effective pharmacologic therapies to date.^[^ ^3^ ^]^ Even though the glucocorticoid therapy given at the early stages of lung inflammation seems to be beneficial for ALI/ARDS,^[^ ^36^ ^]^ its use is associated with a variety of common problems including early osteoporosis, impaired hyperglycemia/blood sugar control, increased risk of immunosuppressive infections and cardiovascular disease.^[^ ^37^ ^]^ A number of nanodevices in the developing pipeline, such as the peptide‐GNP hybrids,^[^ ^38^ ^]^ polydopamine nanoparticles, luminol‐conjugated *β*‐cyclodextrin nanoparticle, and porous Se@SiO2 nano‐spheres, have shown promising effectiveness in reducing inflammation and protecting the lungs from injuries in various ALI animal models.^[^ ^39^ ^]^ Unfortunately, none of them are ready to be translated into clinical uses.

With the drug repurposing strategy, we identified that PPIs have a similar mechanism of action to the anti‐inflammatory nanodevice P12 (Figures 1 and 2). We have validated the anti‐inflammatory effects of PPIs in vitro and the potential new use as the treatment of ALI/ARDS in mice (Figures 3 and 4 and Figure S6, Supporting Information). These studies provided solid evidence that PPIs could be a new therapy for ALI/ARDS when intratracheally administrated. However, inhibition of V‐ATPases systemically by PPIs may lead to some undesired side effects because V‐ATPase is expressed in various cell types (e.g., epithelial cells and macrophages) and responsible for many (patho)physiological functions, including membrane transport, protein degradation, sperm maturation, and bone resorption.^[^ ^40^ ^]^ In fact, long‐term use of PPIs has been associated with some side effects, including gastric carcinoids, reduced absorption of minerals and vitamins, pneumonia, fractures, intestinal infections, hypomagnesemia, chronic kidney disease, and cardiovascular events.^[^ ^41^ ^]^ Therefore, targeted delivery of PPIs to specific cells and organs to control the inflammatory response becomes quite important.

As the alveolar macrophages play a key role in the pathogenesis of ALI/ARDS,^[^ ^42^ ^]^ the alveolar macrophage targeted PPIs would be ideal to specifically regulate lung inflammation at the early stage of ALI/ARDS. This can be easily achieved by formulating PPIs with nanocarriers for macrophage targeting.^[^ ^43^ ^]^ We chose liposomes as the nanocarrier to encapsulate PPIs (Figure 5a,b) because liposomes are well‐characterized delivery systems to encapsulate both hydrophobic and hydrophilic drugs, and can be fast translated into clinical uses as many liposome‐based therapeutics have been FDA approved.^[^ ^44^ ^]^ The nanoform of PPIs (e.g., Nano‐OM) can effectively target lung macrophages, reduce lung inflammation and protect lungs from acute injuries in an LPS‐induced ALI mouse model, where the dose of Nano‐OM was 100‐times lower than molecularly administered OM (Figures 6, 7 and Figure S6, Supporting Information). Altogether, the nano‐enabled drug repurposing of PPIs combined with the clinically used delivery nanocarriers demonstrates an innovative approach to fast develop novel anti‐inflammatory targeted nanodrugs for the treatment of ALI/ARDS.

## 4 Conclusion

Based on our previous discovery of the anti‐inflammatory peptide‐GNP hybrid P12 and understanding its novel mechanisms of actions, we further adapted RNA‐Seq technique and CMAP analysis to identify the clinically used PPIs and enabled its new application in attenuating the TLR activation‐mediated inflammatory responses in macrophages. While all clinically available PPIs were found to have the anti‐inflammatory activity, we particularly confirmed that OM was effective in inhibiting the activation of NF‐*κ*B/AP‐1 and IRF in TLR4 signaling cascades and reducing pro‐inflammatory cytokine production, including IL‐6, TNF‐*α*, and MCP‐1, in THP‐1 cell‐derived macrophages and human PBMC. This inhibitory activity was mainly through the blockage of the endosomal acidification like P12. Accordingly, OM was able to inhibit other endosomal TLRs, such as TLR3 and 7/8, but had no effects on the cell surface TLR2 signaling. To enhance the targeting ability of OM to pulmonary macrophages, liposomal nanocarriers were employed to formulate OM into Nano‐OM. This nano‐formulated OM not only exhibited potent inhibitory activity in vitro but also had enhanced macrophage targeting capability in vivo. In an LPS‐induced ALI mouse model, Nano‐OM demonstrated effective anti‐inflammatory activity on reducing cell infiltration and cytokine production, and protecting lung from injuries. This study provided a nano‐enabled drug repurposing strategy for seeking new clinically available pharmacological agents to treat ALI/ARDS. It also aided to fast translation of novel targeted nano‐drugs for urgent clinical applications to combat human diseases lacking an effective cure, such as ALI/ARDS.

## 5 Experimental Section

### Materials

The TLR agonists LPS (from *Escherichia coli* serotype), high molecular weight PolyI/C, resiquimod (R848), and Pam3CSK4 were purchased from InvivoGen (San Diego, CA, USA). The OM was obtained from Sigma‐Aldrich (Sant‐Louis, MO, USA). They were dissolved in dimethyl sulfoxide (DMSO) (Sigma‐Aldrich, Sant‐Louis, MO, USA) as the stock solution and stored at −20 °C prior to use. The egg yolk lecithin and cholesterol were from Sangon Biotech (Shanghai, China). DiD perchlorate was from Solarbio Science & Technology (Beijing, China). Other chemicals were obtained from Sigma‐Aldrich unless specified. The antibodies against *β*‐actin (#8457S), GAPDH (#2118S), phosphorylated IKK*α*/*β* (p‐IKK*α*/*β*, #2697), p65 (p‐p65, #3033S), I*κ*B*α* (p‐I*κ*B*α*, #2859), and IRF3 (p‐IRF3, #4947S), and total IRF3 (t‐IRF3, #10949S) were obtained from Cell Signaling Technology (Boston, MA, USA). The HRP‐labeled anti‐rabbit (#7074S) or anti‐mouse (#7076S) antibodies were from Cell Signaling Technology (Boston, MA, USA). Viability dye (#L34962) was from Invitrogen (Grand Island, NY, USA). Fluorochrome‐labeled antibodies against mouse CD45 (#563410), Gr1 (#553126), and CD11c (#562782) were from BD Biosciences (San Diego, CA, USA); F4/80 (#123110), and CD11b (#101215) were from BioLegend (San Diego, CA, USA).

### Synthesis and Characterization of the Peptide‐GNP Hybrid P12 and Nano‐OM

The synthesis GNP (13 nm) and P12 was based on the previous work.^[^ ^8^ ^]^ The P12 was prepared by mixing one volume of the peptide CLPFFD (Jietai Inc., Nanjing, China) stock solution (1 mM) with ten volumes of GNP solution at room temperature with overnight incubation. The unbound peptide ligands were removed by high‐speed centrifugation (at 4 °C and 14 000 rpm for more than 30 min) and washing with PBS 3 times.

Nano‐OM was prepared by the thin film hydration method. Briefly, 180 mg of egg yolk lecithin, 60 mg of cholesterol, and 10 mg of OM were dissolved in 10 mL chloroform. The solution was evaporated at 37 °C under reduced pressure to form a uniform lipid film by rotary evaporation. The lipid film was then hydrated at 37 °C with 15 mL PBS (pH 7.4). The lipid vesicles were dispersed and reshaped into uniform liposomal vesicles (Nano‐OM) by ultrasound and extrusion, followed by centrifugation for purification. The concentration of loaded OM in the liposomes was analyzed by methanol rupture method with UV–Vis spectroscopy (U‐3900, Hitachi, Tokyo, Japan). The drug loading efficiency was calculated to be 0.57 ± 0.07%, and the encapsulation efficiency was 7.97±0.9%.

All nanoparticles were filtrated through a syringe filter (0.22 µm, Milipore, Billerica, MA, USA) and processed in a biosafety cabinet to ensure their sterilization for cell culture use and animal studies.

The size of the Nano‐OM was measured using a transmission electron microscope (HT7700, Hitachi, Tokyo, Japan) with an accelerating voltage of 80 kV. The hydrodynamic diameter of the Nano‐OM was determined by dynamic light scattering on a Zetasizer instrument (Nano ZS, Malvern, Worcestershire, UK).

### Culture of Human Monocytic Cells

The human monocytic cell line THP‐1 (from ATCC, Rockefeller, MD, USA) was cultured in the complete RPMI 1640 medium (Gibco, Grand Island, NY, USA) containing 10% fetal bovine serum (Gibco, Grand Island, NY, USA), 2 mM L‐glutamine and 1 mM sodium pyruvate (Gibco, Grand Island, NY, USA) with 5% CO_2_ at 37 °C. The THP‐1 reporter cells were purchased from InvivoGen (San Diego, CA, USA): THP‐1‐XBlue and THP‐1‐Dual. The complete culture medium was supplemented with 200 mg mL^−1^ Zeocin (InvivoGen, San Diego, CA, USA) for the THP‐1‐XBlue cells, whereas 100 mg mL^−1^ Zeocin and 10 mg mL^−1^ blasticidin (InvivoGen, San Diego, CA, USA) were added to the complete medium for the THP‐1‐Dual cells to maintain the selection pressure. To differentiate THP‐1 cells into macrophage‐like cells, they were seeded into a 96‐well (1 × 10^5^ cells well^−1^), 24‐well (5 × 10^5^ cells well^−1^), or 12‐well (2 × 10^6^ cells well^−1^) plate and stimulated with 50 ng mL^−1^ phorbol myristate acetate (Sigma Aldrich, Sant‐Louis, MO, USA) for 24 h, followed by PBS washing and resting for 2 days before experiments. For TLR 7/8 stimulation, THP‐1 monocytes or reporter cells were directly seeded in 96‐well plate (1 × 10^5^ cells well^−1^) followed by stimulation with R848 for 24 h.

Cells were stimulated with different TLR agonists: LPS (TLR4), PolyIC (TLR3), Pam3CSK4 (TLR1/2), and R848 (TLR7/8), and treated with various PPIs or Nano‐OM for 24 h; the culture media were collected for reporter cell assay or stored at −80 °C prior to the cytokine analysis.

### Cell Viability Assay

The viability of THP‐1 cells was measured by MTS assay (Promega, Madison, WI, USA). After 24 h of PPIs or Nano‐OM treatment, the MTS reagent (20 µL well^−1^) was directly added to the 96‐well culture plate, which was incubated at 37 °C for about 2 h. The absorbance at 490 nm was then recorded on a microplate reader (TECAN, Mannedorf, Zurich, Switzerland) in comparison with the untreated control (as 100%).

### NF‐*κ*B/AP‐1 and IRF Reporter Assay

THP‐1‐XBlue reporter cells were used to study NF‐*κ*B/AP‐1 activation. After stimulation and treatments, cell culture medium (20 µL) from each well (96‐well plate) was mixed with 180 µL QUANTI‐Blue solution (InvivoGen, San Diego, CA, USA) in a 96‐well flat bottom plate, which was incubated at 37 °C for 1 or 2 h for the color development. The color change was quantified by the absorption measurement at 655 nm on a microplate reader (TECAN, Mannedorf, Zurich, Switzerland). For the analysis of IRF activation, the THP‐1‐Dual cells were used. After treatments, the culture medium (10 µL) of each sample was transferred into a 96‐well white flat‐bottom plate; and the IRF activation was assessed by measuring the luciferase luminescence on a microplate reader (TECAN, Mannedorf, Zurich, Switzerland) with automated injection of QUANTI‐Luc assay solution (50 µL well^−1^) (InvivoGen, San Diego, CA, USA).

### Human PBMC Isolation and Culture

The collection and use of human PBMC from four healthy adult volunteers with mixed genders were approved by the University of British Columbia Clinical Research Ethics Board (H04‐034), and before each blood draw, written informed consent from each individual was obtained. The blood was collected into the heparin‐coated anticoagulation tube, transferred to centrifuge tubes, and mixed with PBS solution to suspend the cells. The PBMC was isolated by density gradient centrifugation on Ficoll‐Paque Plus (Sigma‐Aldrich, Sant‐Louis, MO, USA). The collected mononuclear cells were centrifuged and washed with PBS three times and resuspended in the complete RPMI‐1640 medium. Cells were plated in a 24‐well plate (5 × 10^5^ cells well^−1^) and rested for 1 h prior to experiments. They were then treated with LPS and OM for 4 h and 24 h, and the culture medium was collected for cytokine analysis.

### RNA Extraction and RNA‐Seq Analysis

Total RNA of THP‐1 cell‐derived macrophages treated with OM (50 mg mL^−1^) or P12 (100 nM) in the presence of LPS (10 ng mL^−1^) for 4 h was extracted using RNeasy mini kit (Qiagen, Hilden, Germany). The concentrations of total RNA samples were determined by a Nanodrop Lite Spectrophotometer (Thermo Scientific, Waltham, MA, USA) and their RNA integrity was assessed by an Agilent 2100 Bioanalyzer (Agilent Technologies, Palo Alto, CA, USA). The RNA‐Seq was performed on Illumina Novaseq 6000 platform with service provided by Novogene Co. Ltd. (Beijing, China).

The bioinformatics analyses were done in the lab as follows. Principal component analysis was performed using the R built‐in function procomp. The DESeq2 R package (1.26.0) was used for differential expression analysis.^[^ ^45^ ^]^ The P‐values were adjusted using Benjamini and Hochberg's approach for controlling the false discovery rate. Genes with an adjusted *P*‐value < 0.05 and |Log_2_FC|>1 were assigned as differentially expressed. The “apeglm” shrinkage estimators were used for LFC shrinkage.^[^ ^46^ ^]^ Among genes with adjusted *P*‐value < 0.05, those with Log_2_FC > 0 were screened as up‐regulated while Log_2_FC < 0 as down‐regulated. The heatmap R package was used to generate heatmaps of differentially expressed genes between groups. The Venn diagram was obtained using the online tool (http://bioinformatics.psb.ugent.be/webtools/Venn/). Pathway analysis was performed by gene set enrichment analyses of differentially expressed genes using KEGG pathway database with Cluster Profiler R package,^[^ ^47^ ^]^ and top 10 significantly enriched pathways were presented.

### Confocal Microscopic Imaging

THP‐1 cells (2 × 10^5^ cells) were seeded in a 20‐mm glass bottom dish (NEST, Wuxi, China) and differentiated into macrophages. They were incubated with pHrodo red‐ and fluorescein‐labeled 10000 MW dextran (10 µg mL^−1^) (Thermo Fisher Scientific, Waltham, MA, USA) for 1 h before OM (500 µg mL^−1^) or chloroquine (30 µM) treatment for 3.5 h. Cells were then washed 3 times with PBS and imaged on a confocal microscope (LSM900, Leica, Wetzlar, Hessen, Germany). The fluorescence intensity of pHrodo red (ex: 565 nm; em: 585 nm) and fluorescein (ex: 488 nm; em: 525 nm) in the cells was quantified by Image J software (NIH, Bethesda, MD, USA). For each condition, at least 30 cells from 3 independent experiments were quantified to obtain the intensity ratio of fluorescein to pHrodo red.

To examine the cellular uptake of Nano‐OM, THP‐1 cell‐derived macrophages were treated with DiD (Solarbio, Beijing, China)‐labeled liposomes for localizing Nano‐OM in the cells, and stained with 25 µM DiO (Beyotime, Shanghai, China) for 20 min for labeling the cell membranes and DAPI (Beyotime, Shanghai, China) for 5 min for labeling the nucleus; the fluorescence images were acquired on a confocal microscope (LSM900, Leica, Wetzlar, Hessen, Germany).

### Immunoblotting Analysis

The THP‐1 cell‐derived macrophages were stimulated with LPS (10 ng mL^−1^) with or without OM, liposomes, and Nano‐OM treatment for different time periods. The cells were lysed with ice‐cold RIPA buffer (Thermo Fisher Scientific, Waltham, MA, USA) supplemented with Halt protease and phosphatase inhibitor cocktail (Thermo Fisher Scientific, Waltham, MA, USA), and the total protein concentration was quantified by the Bradford assay kit (Coomassie Plus, Thermo Fisher Scientific, Waltham, MA, USA) and adjusted accordingly. The proteins were separated by 10% SDS‐PAGE and transferred to a PVDF membrane (Immobilon FL, Millipore, Billerica, MA, USA). The membranes were blocked with 5% bovine serum albumin (Genview, Houston, TX, USA) in the TBS buffer (Solarbio, Beijing, China) containing 0.1% Tween 20 (Sangong Biotech, Shanghai, China) for 1 h at room temperature, followed by blotting with various primary antibodies at 4 °C overnight. They were then washed and blotted with HRP‐conjugated anti‐rabbit or anti‐mouse secondary antibody for 1 h at room temperature; the protein bands were imaged using the chemiluminescence method (ECL, Millipore, Billerica, MA, USA) on a ChemiDoc MP imaging system (Bio‐Rad, Hercules, CA, USA).

### ALI Mouse Model

C57BL/6 wild‐type male mice (6–8 weeks old from SPF Biotechnology Co., Ltd, Beijing, China) were given LPS (10 mg kg^−1^, *E. coli* O111:B4, Sigma, ‐Aldrich, Sant‐Louis, MO, USA) for 24 h through intratracheal injection to establish the ALI mouse model.^[38]^ Liposomes (10 mg kg^−1^) and Nano‐OM (OM: 75 µg kg^−1^) were administered through the same route 1 h before LPS challenge. All animal studies were conducted following the guidelines of the Institutional Animal Care and Use Committee of Tianjin Medical University (TMUaMEC2020004). Mice were sacrificed 24 h after LPS challenge, and the BALF, lung tissue, and blood serum were collected for further analysis.

### BALF Collection and Differential Cell Counting

The BALF collection was performed based on the following procedures. Ice cold PBS (0.8 mL) was injected into the lungs through the trachea twice, and the BALF was collected and centrifuged at 1000 rpm for 10 min at 4 °C. The supernatants were stored at −80 °C for the cytokine analysis, whereas the cell pellets were processed with red blood cell lysis buffer (Solarbio, Beijing, China) and resuspended in PBS for cell counting analysis. Aliquots of the cell suspension were stained with Trypan Blue (Solarbio, Beijing, China), and total cells were counted on a hemocytometer. Meanwhile, the cell suspension (50 µL) was centrifuged onto a glass slide using a cytospin (CytoSpin 4, Thermo Fisher Scientific, Waltham, MA, USA), and processed with Liu stain (Solarbio, Beijing, China). The slides were imaged on a microscope for differential cell counting. After the BALF collection, blood was drawn through the heart apex of the mouse.

### Lung Histology and W/D Ratio

The left larger lobe of the lung was processed for hematoxylin and eosin (H&E) staining. The lung injury was scored based on the infiltration of alveolar and interstitial neutrophils, the formation of hyaline membranes, proteinaceous debris, and alveolar septal thickening.^[^ ^48^ ^]^ The remaining lung tissues were weighed before and after the drying process (60 °C, 48 h) to obtain the W/D ratio.

### Cytokine Analysis

Human cytokines IL‐6, IL‐8, TNF‐*α* and MCP‐1, and mouse cytokines IL‐6 and IL‐1*β* were measured by ELISA kits according to the manufacturer's instructions (Invitrogen, Grand Island, NY, USA or R&D system, Minneapolis, MN, USA).

The Luminex‐based Procarta custom 21‐plex assay (Invitrogen, Grand Island, NY, USA) was used to analyze multiple cytokines/chemokines from human PBMC samples. Assays were read on a Luminex 200 total system running MasterPlex (MiraiBio, Alameda, CA, USA) software. The 21 cytokines quantified in the assay included: TNF‐*α*, M‐CSF, MCP‐3, MCP‐1, MIP‐1*β*, MIG, IP‐10, IL‐6, IL‐23, IL‐18, IL‐12p70, IL‐12p40, IL‐1RA, IL‐1*β*, IL‐1*α*, IFN‐*γ*, IFN‐*β*, IFN‐*α*, GRO‐*α*, GM‐CSF, ENA78. The MFI was interpolated from the standard curve using a five‐parameter logistic formula. Selected cytokines were validated by ELISA (Invitrogen, Grand Island, NY, USA).

### Flow Cytometry Analysis

DiD (Solarbio, Beijing, China) labeled liposomes were intratracheally injected into mice, and the BALF and the lung single cell suspension were collected 24 h later. They were stained with viability dye and various fluorochromes labeled antibodies, including CD45, Gr1, CD11b, F4/80, and CD11c, for flow cytometry analysis. The cells were analyzed on the LSRFortessa X30 (BD, San Diego, CA, USA) flow cytometer, and the data was processed using FlowJo software (TreeStar, Ashland, OR, USA).

### Statistical Analysis

The GraphPad Prism7.0. was used for statistical analysis. All data were expressed as means ± SEM, and *P* < 0.05 was considered statistically significant. For the comparison between two groups, student *t*‐test was used, while one way ANOVA with Bonferroni post‐test was applied for multiple comparison among different groups.

## Conflict of Interest

The authors declare no conflict of interest.

## Supporting information

Supporting Information

## Data Availability

The data that support the findings of this study are available from the corresponding author upon reasonable request.
