# Supplementary material for: Nano‐Enabled Reposition of Proton Pump Inhibitors for TLR Inhibition: Toward A New Targeted Nanotherapy for Acute Lung Injury
Source: Adv Sci (Weinh). 2021 Nov 23;9(3):2104051. doi: 10.1002/advs.202104051 (PMC8787384; doi:10.1002/advs.202104051)
Supplement: Supplementary file 1 — Supporting Information [file ADVS-9-2104051-s001.pdf]

WILEY-VCH

ADVANCED SCIENCE
Open Access

# Supporting Information

for *Adv. Sci.*, DOI: 10.1002/advs.202104051

# Nano-enabled reposition of proton pump inhibitors for TLR inhibition: toward a new targeted nanotherapy for acute lung injury

*Liya Sun, Yuan Liu, Xiali Liu, Rui Wang, Jiameng Gong, Aabida Saferali, Wei Gao, Aying Ma, Huiqiang Ma, Stuart E. Turvey, Shan-Yu Fung**, *and Hong Yang**

# Supporting Information

**Nano-enabled reposition of proton pump inhibitors for TLR inhibition: toward a new targeted nanotherapy for acute lung injury**

*Liya Sun, Yuan Liu, Xiali Liu, Rui Wang, Jiameng Gong, Aabida Saferali, Wei Gao, Aying Ma, Huiqiang Ma, Stuart E. Turvey, Shan-Yu Fung* *, *Hong Yang**

# Supporting Information

**Nano-enabled reposition of proton pump inhibitors for TLR inhibition: toward a new targeted nanotherapy for acute lung injury**

*Liya Sun, Yuan Liu, Xiali Liu, Rui Wang, Jiameng Gong, Aabida Saferali, Wei Gao, Aying Ma, Huiqiang Ma, Stuart E. Turvey, Shan-Yu Fung* *, *Hong Yang**

L. Sun, Y. Liu, R. Wang, J. Gong, H. Ma, H. Yang
School of Biomedical Engineering and The Province and Ministry Co-Sponsored Collaborative Innovation Center for Medical Epigenetics
Tianjin Medical University
No. 22 Qixiangtai Road, Heping district, Tianjin 300070, China
E-mail: hongyang@tmu.edu.cn

X. Liu, W. Gao, A. Ma
Department of Pulmonary and Critical Care Medicine
Shanghai General Hospital
Shanghai Jiao Tong University School of Medicine
No. 650 Xinsongjiang Road, Shanghai 201620, China

A. Saferali
Channing Division of Network Medicine
Brigham and Women's Hospital
Harvard Medical School
181 Longwood Avenue, Boston, MA02115, USA

S.E. Turvey
BC Children's Research Institute
University of British Columbia
950 West 28th Avenue, Vancouver, BC V5Z 4H4, Canada

S.Y. Fung
Department of Immunology, Key Laboratory of Immune Microenvironment and Disease (Ministry of Education), and The Province and Ministry Co-Sponsored Collaborative Innovation Center for Medical Epigenetics
School of Basic Medical Science
Tianjin Medical University
No. 22 Qixiangtai Road, Heping district, Tianjin 300070, China
E-mail: shanefung@tmu.edu.cn

Keywords: bioactive nanoparticles, drug repurposing, acute lung injury, inflammation, macrophages, Toll-like receptors, proton pump inhibitors

**List of contents for supporting information**

**1. Supplementary methods**

**1.1. Cell culture and treatments**

**1.2. Acute lung injury murine model**

**1.3. Immunoblotting analysis**

**2. Supplementary table**

**Table S1:** List of top five drug compounds having similar mechanisms of action to P12 from CMAP analysis

**3. Supplementary figures**

**Figure S1:** The viability of PBMC treated with OM

**Figure S2:** The inhibitory effect of different PPIs on TLR4 signaling pathway in THP-1 reporter cell-derived macrophages

**Figure S3:** The effect of OM on different inflammatory signaling pathways in THP-1 reporter cell-derived macrophages

**Figure S4:** Inhibition of OM on the production of cytokines under LPS stimulation in human bronchial epithelial BEAS-2B cells

**Figure S5:** The effect of OM on TLR3 signaling in A549 reporter cells

**Figure S6:** The protective effect of OM on the LPS-induced ALI mouse model

**Figure S7:** Inhibition of Nano-OM on TLR3 and TLR7/8 signaling in THP-1 reporter cells and their derived macrophages

## 1. Supplementary methods

### 1.1. Cell culture and treatments

The human lung bronchial epithelial (BEAS-2B) cell line (purchased from Cell Bank of Chinese Academy of Sciences, Shanghai, China) was cultured in DMEM medium (Gibco, Grand Island, NY, USA) with 10% FBS (Gibco, USA) with 5% $CO_2$ at 37 ℃. Human lung adenocarcinoma reporter cells (A549-Dual, Grand Island, NY, from InvivoGen) were cultured in DMEM medium (Gibco, USA) containing 10% FBS (fetal bovine serum, Gibco, USA), Zeocin (100 μg/mL) (InvivoGen, USA), Blasticidin (10 μg/mL) (InvivoGen, USA), Normcin (100 μg/mL) (InvivoGen, USA) and 1% penicillin-streptomycin (100 U) （Gino Biomedical Technology Co., Ltd, Shenzhen, China) at 37°C with 5% $CO_2$ in an incubator. Cells were passaged when they reach about 80% confluence. The PPIs lansoprazole, pantoprazole and rabeprazole were from Sigma-Aldrich.

BEAS-2B and A549-Dual cells were seeded ($1.5\times10^4$ cells/well) in a 96-well plate 24 h prior to the experiments. Cells were treated with various PPIs in the presence of different TLR ligands for 24 h, and the culture media were collected and centrifuged at 14,000 rpm, 4 °C for 30 min before reporter cell assay and the cytokine analysis by ELISA following the manufacturer’s instructions.

To measure the NF-κB/AP-1 activation of A549-Dual cells, the supernatants (20 μL) were incubated with QUANTI-Blue solution (180 μL) in a 96-well flat-bottom plate at 37°C for 1-2 h to allow color development, and the absorbance at 655 nm wavelength was measured on a plate reader (TECAN, Mannedorf, Zurich, Switzerland)). For the analysis of IRF activation of A549-Dual cells, the supernatants (10 μL) were added into a 96-well white plate (flat-bottom) and the luciferase activities were measured using a luminometer (TECAN, Switzerland) by auto-injection of QUANTI-Luc solution (50 μL per well).

The viability of PBMC, BEAS-2B cells, A549 Dual cells and the THP-1 reporter cell-derived macrophages upon different PPI (all purchased from Sigma-Aldrich, USA) treatments for 4 h or 24 h was measured by MTS assay (Promega, Madison, WI, USA) following the manufacturer’s instructions. The MTS assay was conducted by measuring the absorbance at 490 nm on a microplate reader (TECAN, Switzerland).

### 1.2. Acute lung injury murine model

C57BL/6 male mice (6-8 weeks old from Shanghai Laboratory Animal Co. Ltd, CHN) were used to establish the ALI mouse model by intranasal administration of LPS (E-coli O111:B4, Sigma, USA). All procedures were performed after intraperitoneal injection of 1% sodium pentobarbital anesthesia (45 mg/kg). Clinically used Omeprazole Sodium for Injection (OM, Losec, AstraZeneca, UK) (10 mg/kg) or PBS (as the negative control) was intratracheally administered 1 h prior to LPS challenge (10 mg/kg). Mice were sacrificed 24 h after LPS challenge for the evaluation of lung inflammation and injury.

### 1.3. Immunoblotting analysis

The THP-1 cell-derived macrophages were stimulated with high molecular weight Poly I/C (50 μg/mL, InvivoGen, USA) with or without OM (50 μg/mL, Sigma, USA) and Nano-OM (phospholipids: 5 mg/mL, OM: 30 μg/mL) for various time periods up to 2 h. The cell lysate preparation and immunoblotting experiments were performed using the same procedures described in the main texts.

**2. Supplementary table**

**Table S1:** List of top five drug compounds having similar mechanisms of action to P12 from CMAP analysis.

| Rank | Compound name and cell line | Similarity mean | N | Enrichment score | P-value | Application/MOA |
|---|---|---|---|---|---|---|
| 1 | Lanatoside C-MCF7 | 0.679 | 3 | 0.942 | 0.00026 | $Na^+/K^+$-ATPase inhibitor |
| 2 | Digoxin-MCF7 | 0.734 | 2 | 0.98 | 0.0007 | $Na^+/K^+$-ATPase inhibitor |
| 3 | Proscillaridin-MCF7 | 0.78 | 2 | 0.977 | 0.00087 | $Na^+/K^+$-ATPase inhibitor |
| 4 | Monensin-MCF7 | 0.65 | 3 | 0.919 | 0.00112 | ionophore |
| 5 | Helveticoside-MCF7 | 0.635 | 3 | 0.916 | 0.0018 | $Na^+/K^+$-ATPase inhibitor |

**Note:** Signature genes (differentially expressed genes) of P12 activity on PBMC were used as query to seek compounds with similar activities of P12 from CMAP database. Compounds with positive similarity mean indicate that they have similar transcriptome changes in comparison with those induced by the P12 treatment in PBMC. The compounds used in specific cell lines from the CMAP database are shown by the compound name and cell line. N is referred to the number of instances obtained from the corresponding compound. MOA: the mechanism of action(s).

**3. Supplementary Figures**

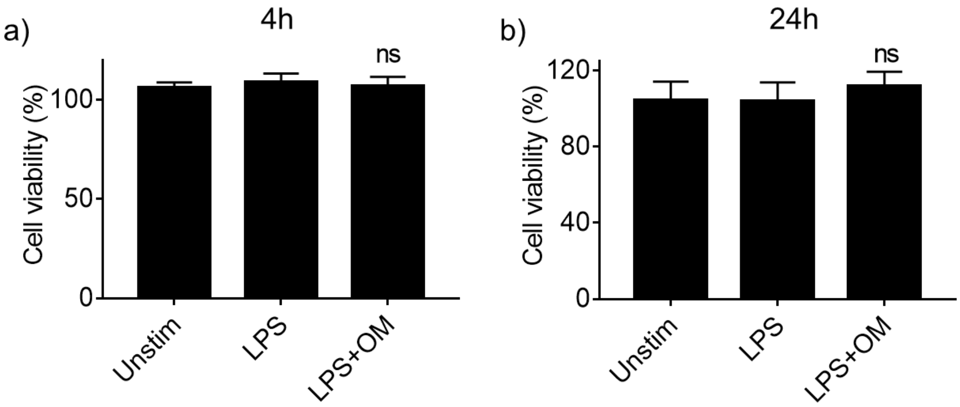


**Figure S1. The viability of PBMC treated with OM analyzed by MTS assay.** OM (50 µg/mL) had no effect on the cell viability of PBMC at 4 h (a) and 24 h (b). LPS: 10 ng/mL, OM: 50 µg/mL, N = 3, ns: not significant.

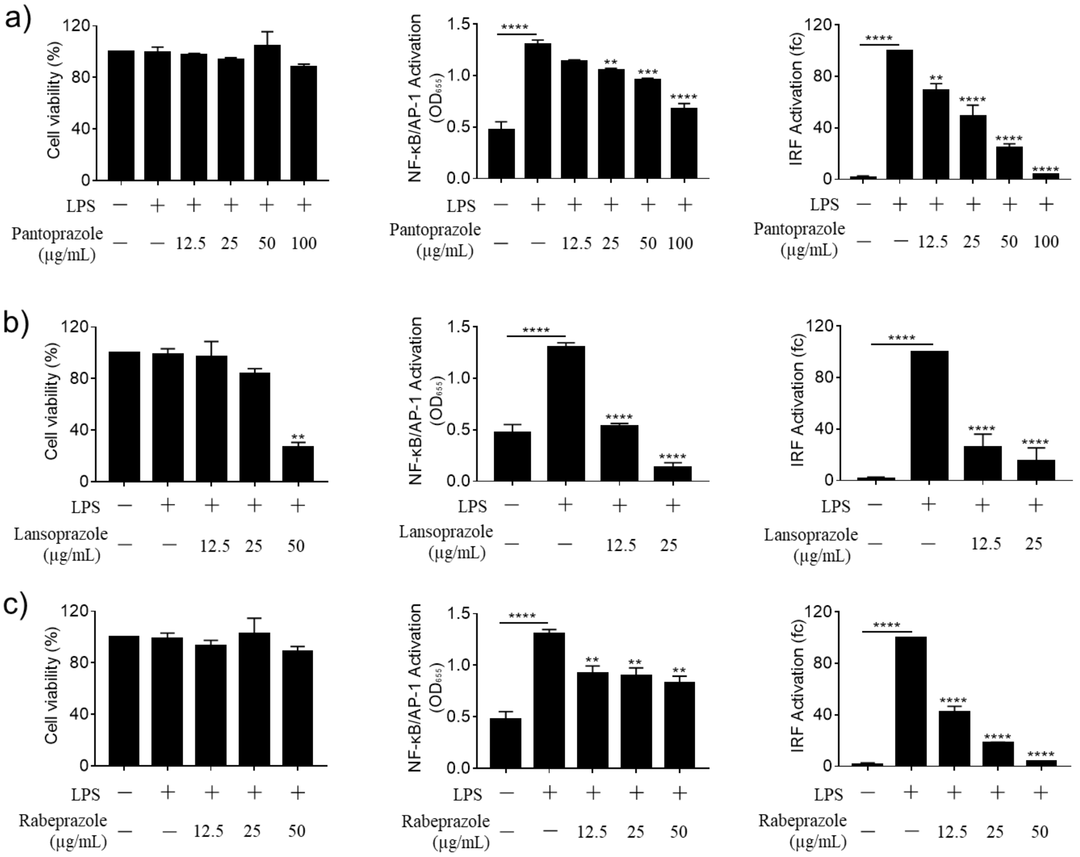


**Figure S2. The inhibitory effect of different PPIs on TLR4 signaling pathway in THP-1 reporter cell-derived macrophages.** a) The effect of pantoprazole at various concentrations (12.5, 25, 50 and 100 μg/mL) on the cell viability (left), and the activation of NF-κB/AP-1 (middle) and IRF (right) triggered by LPS. b) The effect of lansoprazole at various concentrations (12.5, 25 and 50 μg/mL) on the cell viability (left), and the activation of NF-κB/AP-1 (middle) and IRF (right) triggered by LPS. c) The effect of rabeprazole at various concentrations (12.5, 25 and 50 μg/mL) on the cell viability (left), and the activation of NF-κB/AP-1 (middle) and IRF triggered by LPS. LPS: 10 ng/mL, N = 3, fc: fold changes, $**p < 0.01$, $***p < 0.001$, $****p < 0.0001$.

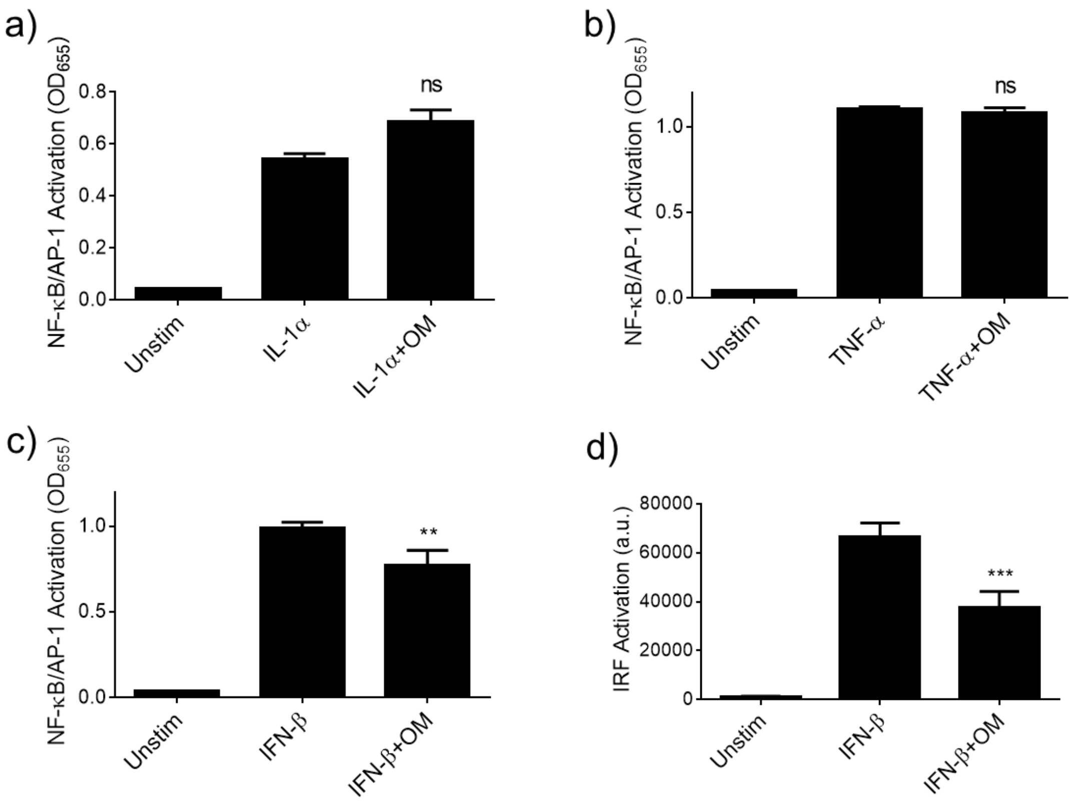


**Figure S3. The effect of OM on different inflammatory signaling pathways in THP-1 reporter cell-derived macrophages.** OM did not inhibit the activation of NF-κB/AP-1 induced by a) IL-1α or by b) TNF-α. However, OM could inhibit the activation of both c) NF-κB/AP-1 and d) IRF induced by IFN-β. OM: 50 μg/mL, IL-1α: 100 ng/mL, TNF-α: 100 ng/mL, IFN-β: 100 ng/mL, N = 3, $^{**}p < 0.01$, $^{***}p < 0.001$, ns: not significant.

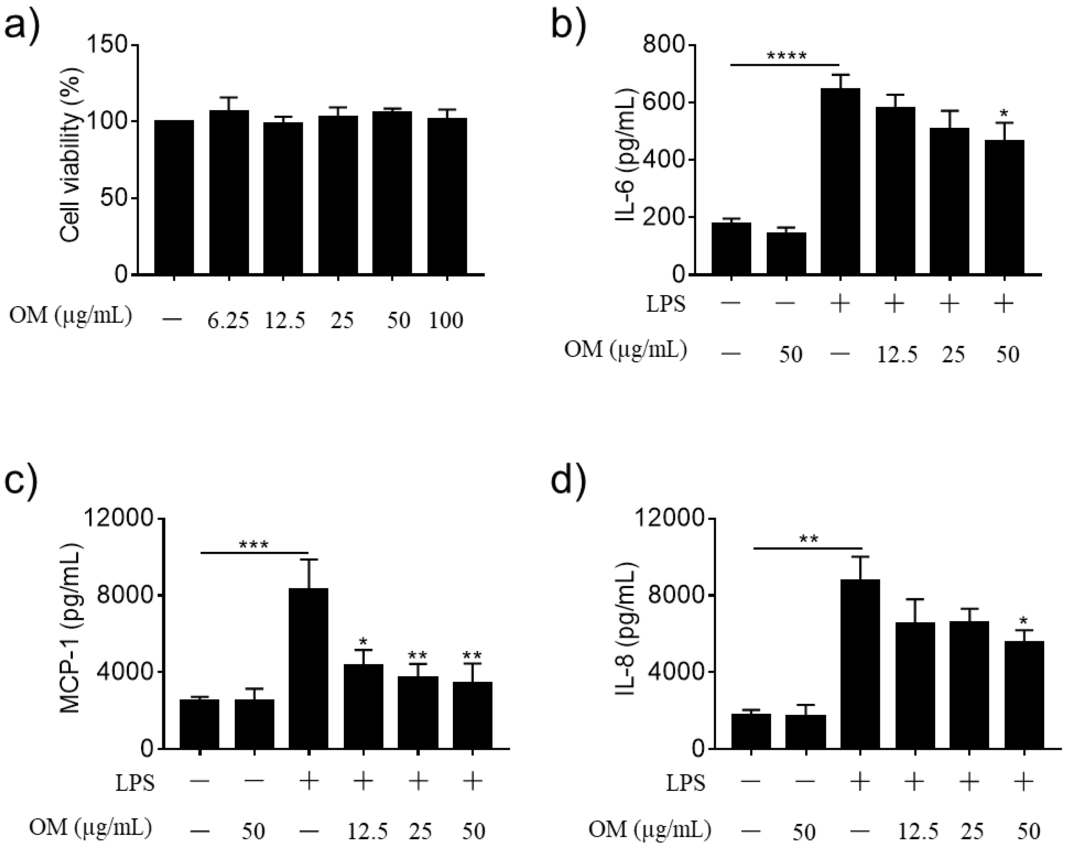


**Figure S4. Inhibition of OM on the production of cytokines under LPS stimulation in BEAS-2B cells.** a) OM at different concentrations had no effect on the cell viability (N = 4). ELISA analyses demonstrated that high concentration(s) of OM inhibited the production of cytokines b) IL-6, c) MCP-1 and d) IL-8 stimulated by LPS, N = 4 for IL-6, N = 3 for MCP-1 and IL-8. LPS = 10 ng/mL, *$p < 0.05$, **$p < 0.01$, ***$p < 0.001$, ****$p < 0.0001$.

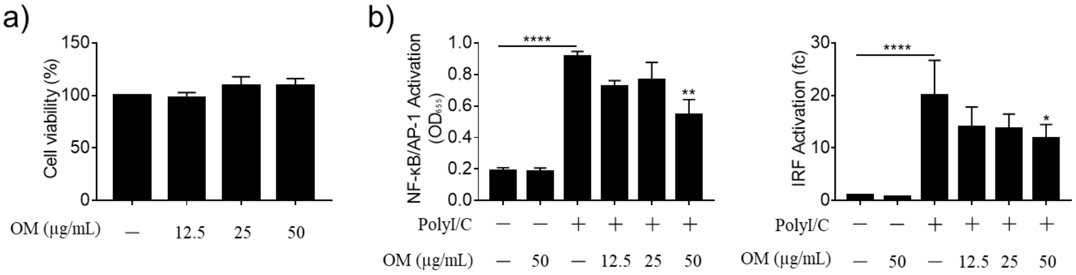


**Figure S5. The effect of OM on TLR3 signaling in A549 reporter cells. a) OM at different concentrations had no effect on the cell viability.** b) OM at the concentration of 50 μg/mL inhibited NF-κB/AP-1 (left) and IRF (right) activation of A549 cells stimulated by Poly I/C (50 μg/mL). N = 3-4, *$p < 0.05$, **$p < 0.01$, ****$p < 0.0001$.

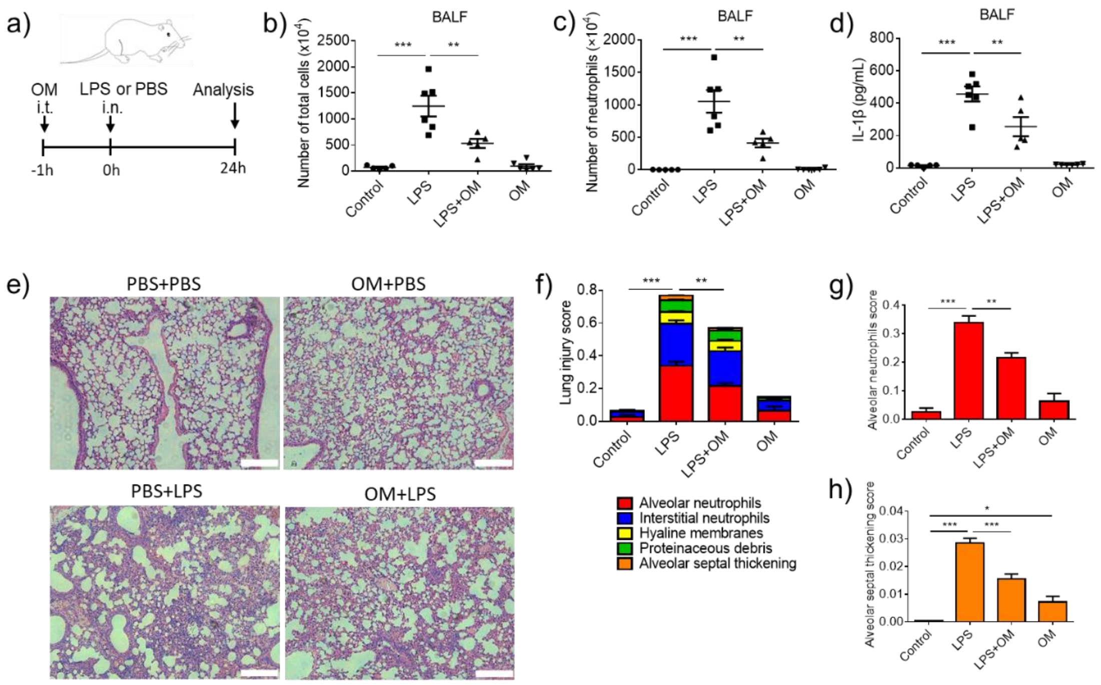


**Figure S6. The protective effect of OM on the LPS-induced ALI mouse model.** a) The scheme of OM pre-treatment in the LPS-induced ALI model. b) The total number of cells in the BALF. c) The neutrophil counts in the BALF. d) Inhibition of the cytokine IL-1β production in the BALF. e) The images of H&E stained lung sections 24 h after LPS stimulation; the scale bar = 200 μm. f) The total lung injury score based on the 5 pathophysiological characteristics. g) Alveolar neutrophils score. h) Alveolar septal thickening score. LPS = 10 mg/kg, OM = 10 mg/kg, N = 5 per group, $*p < 0.05$, $**p < 0.01$, $***p < 0.001$.

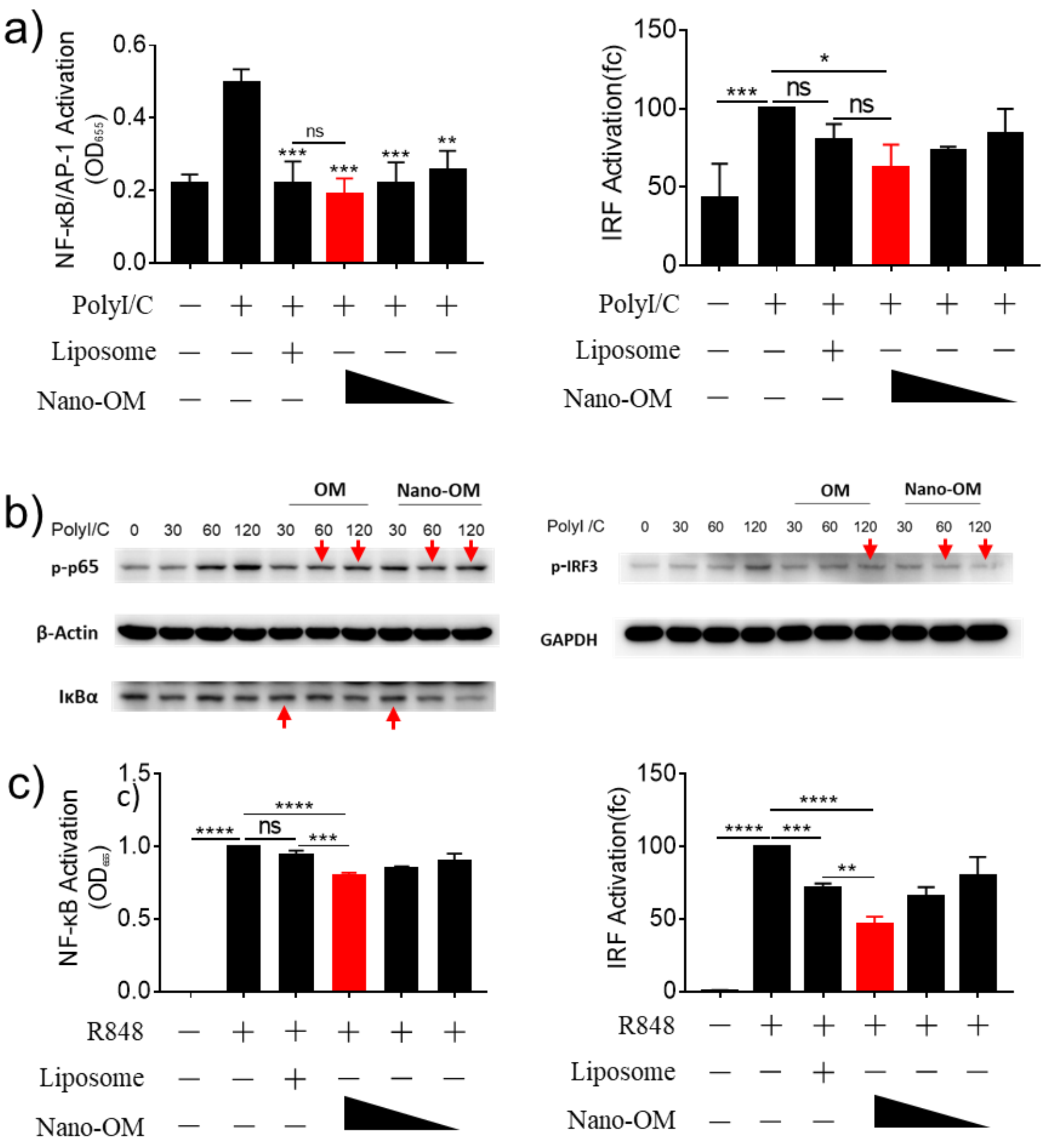


**Figure S7. Inhibition of Nano-OM on TLR3 and TLR7/8 signaling in THP-1 reporter cells and their derived macrophages.** a) Inhibition of NF-κB/AP-1 (left) and IRF activation (right) by Nano-OM upon Poly I/C stimulation. b) Immunoblotting to verify the inhibitory effect of OM (50 µg/mL) and Nano-OM (phospholipids: 5 mg/mL, OM: 30 µg/mL) on the phosphorylation of p65 (p-p65) and degradation of IκBα (left) for NF-κB activation, and the phosphorylation of IRF3 (p-IRF3) for IRF3 activation; the red arrows indicated the differences compared with the Poly I/C stimulation only at the same time point. c) Inhibition of NF-κB/AP-1 (left) and IRF activation (right) by Nano-OM under the R848 stimulation. Poly I/C = 50 µg/mL, R848 = 10 µg/mL, Lipo (phospholipids: 5 mg/mL), Nano-OM (OM: 30, 15, 7.5 µg/mL), N = 3-4, *$p < 0.05$, **$p < 0.01$, ***$p < 0.001$, ****$p < 0.0001$, ns: not significant.
